# Supplementary material for: Evaluating ChatGPT’s Concordance with Clinical Guidelines of Ménière’s Disease in Chinese
Source: Diagnostics (Basel). 2025 Aug 11;15(16):2006. doi: 10.3390/diagnostics15162006 (PMC12385325; doi:10.3390/diagnostics15162006)
Supplement: Supplementary file 1 [file diagnostics-15-02006-s001.zip › Supplement 1.pdf]

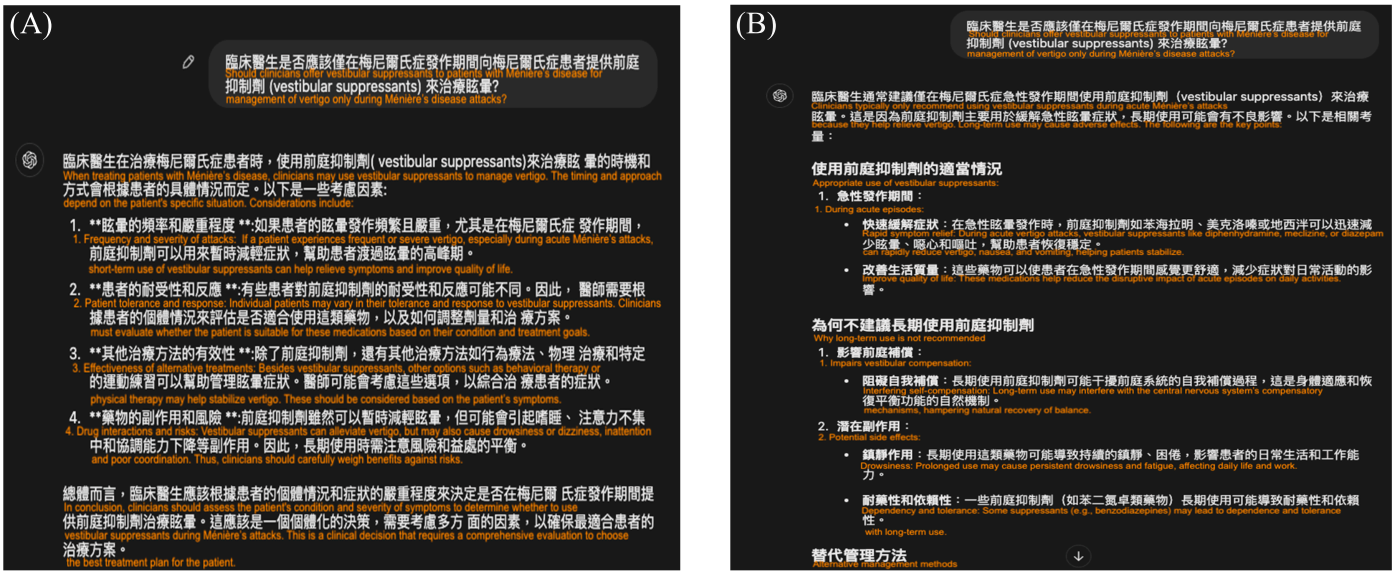

Figure S1A an S1B. An example of ChatGPT responses to the KAS 7 in the AAO-HNS CPG of MD. (A) ChatGPT 3.5; (B) ChatGPT 4.0.

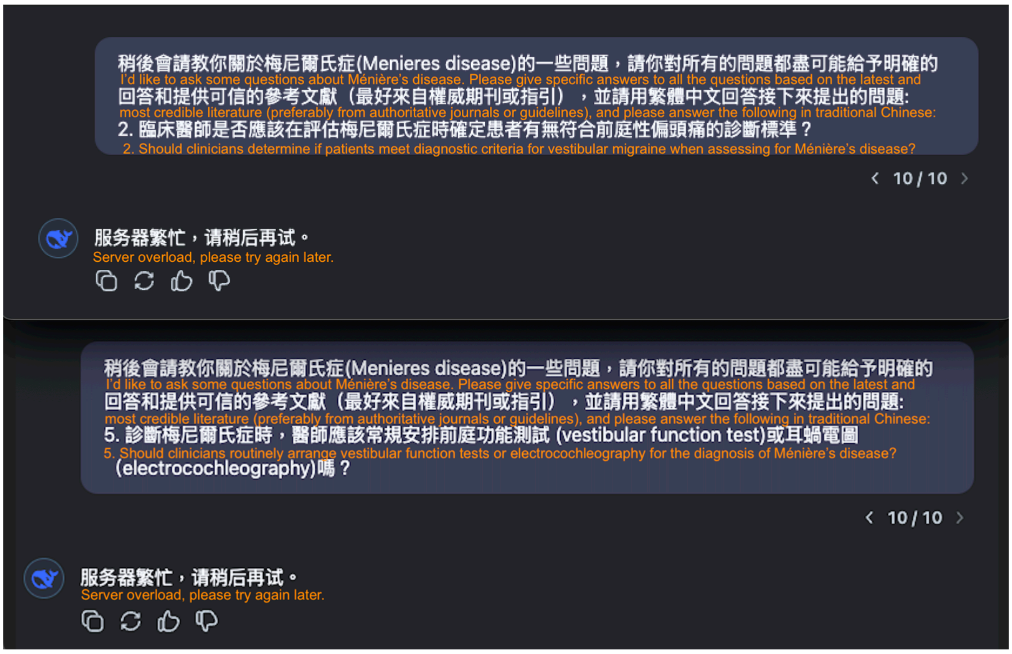

Figure S2. Frequent server overload alerts occurred during queries with DeepSeek
